# The Relation of Chlorids to Edema

**Published:** 1907-05

**Authors:** 


### THE RELATION OF CHLORIDS TO EDEMA.

---

Although several writers had previously suggested the possibility of edema being due, at least in certain conditions, to the presence abnormal quantities of sodium chlorid in the tissues, yet general attention was first called to this possibility by the observations and writings of H. Strauss in Germany and of Widal in France in 1902 and 1903. These clinicians observed a striking correlation between the elimination of chlorids and the development of dropsy in certain cases of nephritis, and brought forward evidence to support the hypothesis that in nephritis the ability of the kidney to excrete sodium chlorid is decreased, and that the retention of this salt in the body causes a corresponding retention of water to dilute the salt to that degree of concentration which is comptaible with the normal osmotic pressure of the blood and tissue fluids. Many clinicians adopted the suggestions of Strauss and Widal as to the treatment of edema by reduction of the chlorids in the food, and soon the literature of all countries was filled with reports on this subject, describing results and giving conclusions greatly at variance with each other. The first excitement having passed, it is now safe to analyze the various reports that thus far have been submitted, and to attempt to draw conclusions with some hope that they may be reasonably correct.*

In the first place, the reports of many competent observers leave us no choice but to conclude that in nephritis, particularly of the parenchymatous type, retention of chlorids often occurs; yet in some typical cases with edema it may not be possible to demonstrate such

1- A full bibliography of this subject will be found in the resume by Martin Kaufmanu, Centrbl. f. d. gesamte Physiol. u. Pathol. des Ses Stoffwechsels, 1906 vii, 487.

retention. Also, patients may show a distinct chlorid retention for some time, and then, without evident reason, the retention may cease. Furthermore, in some cases of interstitial nephritis retention may be as marked as in the parenchymatous forms, and this retention is not always associated with edema. In certain cases of nephritis, the edema will be found to vary directly with the degree of chlorid retention, as in the striking instance reported by Widal and Javal, in which it was possible to cause the edema to appear and disappear almost at will by varying the amount of chlorids in the diet. In other cases, however, even the most careful reduction of the chlorid intake will not appreciably modify the edema, while in perhaps the largest number of cases the amount of edema may be reduced by this means, but not always strikingly or constantly. In other words, the weight of evidence indicates that retention of chlorids frequently is an important feature in the edema of nephritis, but it is not always possible to demonstrate its relation to the edema. Apparenly the power of the kidneys to eliminate chlorids varies from time to time, just as the excretion of albumin and the presence of casts may vary. Probably one important reason for our inability to demonstrate a clear relation between chlorid excretion and edema lies in the great power of the body to retain fluid without the development of edema; for example, in one case of nephritis it was observed that feeding salt led to a marked retention of water, but edema did not begin to appear until the patient's weight showed that twelve pounds of water had been accumulated in the body. This power of retaining water without manifestation of edema makes it impossible to establish a close parallelism between the chlorid excretion and the edema, especially when the cases can not be studied very accurately for long periods of time.

Some observers have thought that the primary trouble in nephritic edema is the retention of water, to which the retention of salt is secondary; but the weight of evidence here seems to be that the chlorid retention is primary and the water retention is secondary. The retention of the chlorids seems to depend entirely on the inability of the kidneys to excrete them. Apparently the retained chlorids are held in the tissues throughout the body, for many analyses have shown a retention of chlorids in the tissues—the so-called histo-retention. The amount of chlorids in the blood is not, and can not be, greatly increased, for water must always accumulate to keep the osmotic concentration of the blood not far from normal. Why and how the chlorids accumulate in the tissues in excessive amounts is one of the unsolved problems.

There seems to be no tendency on the part of those who question the general importance of chlorid retention in the etiology of edema to deny the facts brought forward by Widal and others, which show that in at least some cases this retention is the cause of the accompanying edema. The chief question is: How often does edema depend on chlorid retention, and how often is this factor absent or of minor importance as compared with the other possible causes of edema that may be present in nephritic patients? The answer to this question can not possibly be given on the strength of the information afforded by the discordant reports that are to be found in the literature, but it seems safe to say that chlorid retention is an imporant factor in a very considerable proportion of nephritic edemas. Chlorid retention has also been observed in some cases of dropsy in heart disease, and with some edemas occurring in patients with acute inflammatory disorders, but here it is not so well established that the observed chlorid retention is more than can be explained as due to the retention of the fluid in the edematous parts.

In any case, however, the treatment of renal dropsy by "de-chloridization therapy" seems to be warranted on several grounds. In the first place, of course, comes the indication that in many cases edema is relieved by this means; the amount of chlorids excreted on a chlorid-poor diet being much in excess of the amount taken in, such a diet soon leads to removal of the retained chlorids and with them the retained water. Secondly, there is considerable evidence that chlorids may act injuriously on the diseased kidneys; indeed, excessive quantities of sodium chlorid may cause albuminuria even in healthy individuals, which observation suggests the possibility that the use of oversalted foods may be an important factor in the production of nephritis. It seems to have been commonly found that nephritic patients kept on a chlorid-poor diet show a decreased amount of albumin in the urine, while administration of chlorids causes it to increase. Furthermore, there is abundant evidence that there is no danger in reducing the intake of chlorids to as low a figure as can be done by a carefully selected diet. To reduce the chlorids to such a reasonable minimum, say from 2 to 3 grains a day, no very difficult procedures are necessary, for in eggs, unseasoned meats and unsalted butter we have foods that are nearly salt-free, while the salt content of milk is very low. Bread without salt, fresh water fish, potatoes, rice, fresh vegetables, fruits and chocolate offer a considerable variety of salt-poor foods that make the regulations of a dechloridization diet not intolerable to the patient. It may be added that Widal insists on repeated weighing of the patient, in order to keep track of the degree to which the water is being removed during the treatment.—Editorial, Journal American Medical Association, April 13, 1906.

